# The role of dynamic Lowenstein occupational therapy and cognitive assessment scale for the elderly with Parkinson’s disease

**DOI:** 10.1186/s12877-026-07844-6

**Published:** 2026-06-19

**Authors:** Fatıma Yaman, Fatma Akkoyun Arıkan, Esra Aysu Soylu, Elif Pekşen, Merve Akdeniz Leblebicier, Aysun Özlü, Hasan Hüseyin Gökpınar, Barış Yenen, Vural Kavuncu

**Affiliations:** 1 Department of Physical Medicine and Rehabilitation, Kütahya Health Sciences University, Kütahya, Türkiye; 2 Department of Neurology, Kütahya Health Sciences University, Kütahya, Türkiye

**Keywords:** Parkinson disease, Cognitive dysfunction, Neurocognitive disorders, Mental status and dementia tests

## Abstract

**Background:**

Cognitive impairments in Parkinson’s disease (PD) commonly affect executive function, attention, and visuospatial abilities. These deficits may precede motor symptoms and are often undetected by screening tools such as the Standardized Mini-Mental State Examination (SMMSE). The utility of the Dynamic Lowenstein Occupational Therapy Cognitive Assessment–Geriatric Version (DLOTCA-G) has not been evaluated in older adults with PD. This study aims to investigate the effectiveness of DLOTCA-G in detecting cognitive deficits in PD patients with and without cognitive impairment.

**Methods:**

In this prospective controlled study, 90 participants aged 65 and above were recruited: 30 PD patients with cognitive impairment (PD-CI), 30 PD patients without cognitive impairment (PD-NCI), and 30 healthy controls. All participants underwent cognitive assessment using SMMSE and DLOTCA-G. The total SMMSE score and eight subscales of the DLOTCA-G were analyzed.

**Results:**

Except for the spatial perception score, there was a statistically significant difference between the groups in all DLOTCA-G subscales. The PD-CI group had significantly lower awareness and visuomotor construction scores than the PD-NCI and healthy groups (*p* < 0.01). The PD-NCI group showed significantly lower awareness and visuomotor construction scores than the healthy group (*p* < 0.01). Total SMMSE scores showed a moderate positive correlation with orientation, praxis, visuomotor construction, and thinking operations (*p* < 0.01). In the Parkinson’s group (*n* = 60), SMMSE Orientation correlated strongly with DLOTCA-G Orientation, Visuomotor Construction, and Awareness (*p* < 0.001). SMMSE Attention/Calculation and Language showed moderate associations with praxis and visuomotor domains (*p* ≤ 0.001; Table 4).

**Conclusion:**

The results indicate that DLOTCA-G subscales associated with executive function are impaired in individuals with PD, even in the absence of cognitive impairment. These findings suggest that DLOTCA-G may offer a more differentiated cognitive profile than SMMSE across multiple domains, although its comparative sensitivity and diagnostic accuracy require further validation in studies using comprehensive neuropsychological references.

**Trial registration:**

The study was retrospectively registered (registration number NCT06131619), prospective registration prior to participant enrollment was not completed due to logistical reasons. Protocol adherence was maintained throughout the study.

## Background

Parkinson’s disease is the second most common neurodegenerative disease after Alzheimer’s Disease. The prevalence of Parkinson’s disease in the later stages of life has been reported to be 2–3% in individuals aged 65 or older, and 10% in those aged 80 or older. The slow onset of motor symptoms in people with Parkinson’s disease may lead to nonmotor symptoms, especially cognitive dysfunction, being overlooked [1]. However, cognitive dysfunction occurs in all stages of Parkinson’s disease, including the early stage [2]. Unlike early-stage Alzheimer’s disease, in which cognitive impairments are primarily seen in memory, Parkinson’s disease often affects attention, language, visuospatial skills, and executive functions [3]. Given that the increased relative risk of dementia in people with Parkinson’s disease is directly related to the progression of the disease, it is important to recognize and manage cognitive involvement early, since executive deficits have been shown to interact with neuropsychiatric symptoms and daily functioning in PD-MCI (Parkinson’s disease–related mild cognitive impairment); executive dysfunction is widely recognized as one of the earliest and most clinically meaningful cognitive changes in PD, impairments in planning, cognitive flexibility, and goal directed behavior may precede the onset of dementia and are strongly associated with functional decline, illustrating the importance of assessment tools, such as the DLOTCA-G, that include structured executive domains capable of detecting subtle and clinically relevant cognitive changes beyond global screening measures [4–6].

For this purpose, the Standardized Mini-Mental State Examination (SMMSE) is often used for screening due to the time-consuming nature of neuropsychometric tests. However, research has shown that the SMMSE is insufficient in detecting verbal-visual memory, thought flow, and planning disorders in people with Parkinson’s disease with scores of 23 or above [7]. Therefore, there is a need for a cognitive assessment battery tailored explicitly to the geriatric population that evaluates cognitive function effectively, comprehensively, and efficiently, primarily for individuals with Parkinson’s disease aged 65 or older. The Dynamic Lowenstein Occupational Therapy Cognitive Assessment-Geriatric Version (DLOTCA-G) is an updated performance measurement battery of the Loewenstein Occupational Therapy Cognitive Assessment-Geriatric (LOTCA-G), designed to evaluate basic cognitive skills and visual perception in geriatric patients with neurological deficits. In the geriatric population, it provides an assessment of physical and mental factors through reduced pictorial details, mediation guidelines and multiple-choice questions. Unlike comprehensive neuropsychological batteries recommended by the International Parkinson and Movement Disorder Society for detailed cognitive profiling [8], which may require 1–2 h or more, the DLOTCA-G provides a structured yet clinically feasible evaluation within a moderate administration time [9, 10]. While there are studies in the literature on the usefulness of DLOTCA-G for cognitive evaluation in patients with stroke and spinal surgery, no research has yet examined its effectiveness in older people with Parkinson’s disease [11]. The DLOTCA-G not only assesses cognitive functions but also evaluates how these functions translate into performance in daily life and occupational activities, providing clinically valuable information for individualized occupational therapy planning in patients with Parkinson’s disease [9].

The primary hypothesis of our study is that DLOTCA-G detects subtle cognitive deficits in Parkinson’s disease patients, including those without clinically apparent impairment, more effectively than SMMSE.

The aim of our study was to evaluate the usefulness of DLOTCA-G in detecting cognitive impairment in people with Parkinson’s disease aged 65 or older, both with and without cognitive impairment. Additionally, we sought to examine the relationship between total SMMSE scores and the subscale of DLOTCA-G in these patients.

## Methods

### Participants

The study included three groups:

1. Parkinson’s disease group with cognitive impairment (PD-CI, *n* = 30): Patients aged 65 or older diagnosed with Parkinson’s disease (Hoehn Yahr stage 1–2.5) who scored below 20 points on the SMMSE subscales for individuals with less than 5 years of education history or below 24 points on the SMMSE subscales for individuals with more than 5 years of education history, indicating cognitive impairment.
2. Parkinson’s disease group without cognitive impairment (PD-NCI, *n* = 30): Patients aged 65 or older diagnosed with Parkinson’s disease (Hoehn Yahr stage 1–2.5) who scored 20 points or higher on the SMMSE subscales for individuals with less than 5 years of education history or 24 points or higher on the SMMSE subscales for individuals with education more than 5 years, indicating no clinically significant cognitive impairment.
3. Healthy control group (HC, *n* = 30): Individuals aged 65 or older with no history of neurological or psychiatric disease and SMMSE scores of 29–30.

The Parkinson’s disease groups consisted of patients aged 65 or older diagnosed with Parkinson’s disease by F.A.A. at the Neurology Department, Movement Disorders Polyclinic of Kütahya Health Sciences University. The healthy control group consisted of 30 individuals aged 65 or older who visited the Physical Medicine and Rehabilitation outpatient clinic at Kütahya Health Sciences University.

The neurological examinations were performed by F.A.A., patients with a congenital or central nervous system disease such as stroke or traumatic brain injury, abnormal central nervous system examination findings, history of drug or alcohol abuse, psychiatric history, or family history of psychiatric illness, a Geriatric Depression Scale score of 13 or above were excluded from the study. The Turkish validity and reliability of the scale have been previously established by Ertan T et al. [12].

All participants were informed about the evaluation parameters. Those who agreed to participate in the study and signed the consent forms were included across all three groups.

### Measures

SMMSE: The Standardized Mini-Mental State Examination (SMMSE) is a structured version of the original Mini-Mental State Examination (MMSE; Folstein et al., 1975), developed by Molloy et al. to improve inter-rater and intra-rater reliability through standardized administration and scoring instructions [13, 14]. Like the original instrument, it assesses orientation, memory, attention, calculation, recall, language, motor function, perception, and visuospatial abilities, and consists of items yielding a total score of 30 points. A score between 24 and 30 is considered normal, 18 to 23 indicates mild dementia, and 17 or below suggests severe dementia. The Turkish validity and reliability study of the standardized version was conducted by Güngen et al. [15]. According to Crum RM et al. (1993), scores on the test were significantly associated with educational level; the median score was 29 in individuals with at least 9 years of education, 26 in those with 5–8 years of education, and 22 in those with 0–4 years of education [16]. In the Turkish population these educational-level cut-offs differ by 2–3 points, probably because of sociocultural differences [17]. Scores were obtained from eight sub-scales and recorded. Cognitive impairment was determined using the SMMSE according to participants’ education level, based on Turkish normative data. Individuals with less than 5 years of education: SMMSE score < 20 indicated cognitive impairment. Individuals with 5 years of education or higher: SMMSE score < 24 indicated cognitive impairment. These cut-off scores were applied consistently to classify participants into the PD with cognitive impairment (PD-CI) and PD without cognitive impairment (PD-NCI) groups [15].

It should be emphasized that grouping based on SMMSE scores in this study was performed for research stratification purposes only and does not correspond to a formal diagnosis of Parkinson’s disease–related mild cognitive impairment (PD-MCI). According to the Movement Disorder Society (MDS) Task Force criteria, PD-MCI diagnosis needs a comprehensive neuropsychological assessment across multiple cognitive domains [18].

DLOTCA-G: DLOTCA-G consists of 8 cognitive areas and 24 subtests (Orientation, Visual Perception, Spatial Perception, Praxis, Visuomotor Construction, Thinking Operations, Awareness, and Memory). All sub-scales, except for orientation (scored between 1 and 8 points), are scored from 1 to 4 points. In the present study, the median administration time of the DLOTCA-G was 52 min, indicating a moderate and practical duration for clinical evaluation. The objectives of the assessment are as follows:

- Identify cognitive abilities and disabilities of individuals in various areas.
- Measure learning potential and cognitive changes.
- Recognize thinking strategies.
- Assess the individual’s level of self-awareness, status, and cognitive disabilities.

### Procedure

Occupational therapists evaluated the DLOTCA-G and SMMSE subscales of the participants in a quiet room where external distractions were eliminated. SMMSE was administered by the same occupational therapist (E.P.) to patients with Hoehn-Yahr staging between 1 and 2.5. Based on SMMSE scores, patients were divided into two groups: those with and those without cognitive impairment. DLOTCA-G was administered to both Parkinson’s disease and healthy control groups by a different occupational therapist (E.A.S.)

### Statistical analysis

A preliminary study was conducted with 10 individuals in each group to determine the effect size. Using the visuomotor construction score levels obtained from this study, a power analysis was carried out using G*Power 3.1.9.4 software (Version 3.1.5, University of Kiel, Germany). The analysis concluded that to achieve 90% statistical power with an effect size of f = 0.42, each group should comprise a minimum of 25 participants. As a result of the analysis, a total of 75 patients were required; to allow for a 20% drop-out rate, we recruited 90 patients.

All statistical analyses were performed using SPSS 25.0 (IBM SPSS Statistics 25 software; Armonk, NY: IBM Corp.). Continuous variables were presented as mean ± standard deviation, and median (IQR: 25th − 75th percentiles), and categorical variables were given as numbers and percentages. The Shapiro-Wilk test was used to assess normality. For intergroup comparisons, the Mann-Whitney U test and Kruskal-Wallis Analysis of Variance (with post hoc analysis using the Mann-Whitney U test and Bonferroni correction) were applied. To control for the potential confounding effects of age and education, an ANCOVA was also performed, with group as a fixed factor and SMMSE and DLOTCA-G total scores as dependent variables. Although Kruskal-Wallis and ANCOVA rely on different assumptions, the results were compared to check whether group differences remained consistent in direction and significance. The Chi-square test was used to compare categorical variables. The Spearman correlation coefficient was used to analyze relationships between continuous variables. A p-value of ≤ 0.05 was considered statistically significant. Correlation coefficients were interpreted as follows: 0-0.1 as insignificant, 0.1–0.39 as weak, 0.4–0.69 as moderate, 0.7–0.89 as strong, and above 0.9 as a very strong correlation [19].

## Results

The study was completed with 60 Parkinson’s patients (PD-CI group *n* = 30 [33.3%], PD-NCI group *n* = 30 [33.3%]) and 30 healthy controls (33.3%). Among them, the mean age of the cognitive impairment group (PD-CI) was 73.2 (5.74) years, the mean age of the Parkinson without cognitive impairment (PD-NCI) group was 70.07 (4.93) years, and the mean age of the healthy control group was 71.4 (5.42) years (*p* = 0.108). There was no significant difference between the groups in terms of educational status, Hoehn-Yahr stage, or disease duration (Table 1).

**Table 1:** Demographic and clinical data

		PD-CI	PD-NCI	Healthy control group	Intergroup p
Age	Mean ± S.D.	73.2 ± 5.74	70.07 ± 4.93	71.4 ± 5.42	0.108 (kw = 4.447)
	Med (IQR)	72 (69.75–78.25)	70 (65–74)	70 (66–75)	
Gender	Female	17 (%56.7)	14 (%46.7)	17 (%56.7)	0.669 (cs = 0.804)
	Male	13 (%43.3)	16 (%53.3)	13 (%43.3)	
Education	Primary School	26 (%86.7)	23 (%76.7)	25 (%83.3)	0.519 (cs = 5.192)
	Secondary School	3 (%10)	3 (%10)	1 (%3.3)	
	High School	0 (%0)	2 (%6.7)	2 (%6.7)	
	University	1 (%3.3)	2 (%6.7)	2 (%6.7)	
Hoehn and Yahr	1.00	13 (%43.3)	16 (%53.3)	-	0.32 (cs = 3.505)
	1.50	5 (%16.7)	8 (%26.7)	-	
	2.00	6 (%20)	4 (%13.3)	-	
	2.50	6 (%20)	2 (%6.7)	-	
Duration of the Disease	Mean ± S.D.	3.7 ± 1.66	3.63 ± 1.47	-	1 (z = 0)
	Med (IQR)	4 (2–5)	3 (2–5)	-	

*S.D* Standard Deviation, *Med (IQR)* Median (25th-75th percentiles), *cs* Chi-Square test, *kw* Kruskal Wallis Analysis of Variance, *z* Mann Whitney U test

Upon examining the awareness and visuomotor construction scores, it was found that the PD-CI group had significantly lower scores than the PD-NCI group and the healthy control group (*p* = 0.0001). ANCOVA results indicated that, after controlling for age and education, significant differences between groups were maintained for both SMMSE (F (2,85) = 80.15, *p* < 0.001) and total DLOTCA-G scores (F (2,85) = 35.14, *p* < 0.001). These data suggest that the observed group differences were independent of age and education. Inclusion of the covariates in the model did not alter the significance or direction of the group comparisons. Additionally, the awareness and visuomotor construction scores were significantly lower in the PD-NCI group compared to the healthy control group. Also, the orientation, praxis, and thinking operations scores were lower in the PD-CI group than in the PD-NCI group and the healthy control group (*p* = 0.0001) (Table 2).

**Table 2:** Comparison of DLOTCA-G subscale scores between groups

		PD-CI	PD-NCI	Healthy Control Group	Intergroup p
Orientation	Mean ± S.D.	1.37 ± 0.58	1.95 ± 0.1	1.97 ± 0.11	0.0001* (kw = 40.835) a, b
	Med (IQR)	1.5 (0.97–1.91)	2 (1.88–2)	2 (2–2)	
Visual perception	Mean ± S.D.	3.44 ± 0.38	3.61 ± 0.35	3.66 ± 0.16	0.03* (kw = 7.025) a
	Med (IQR)	3.67 (3.25–3.67)	3.67 (3.67–3.75)	3.67 (3.67–3.67)	
Spatial perception	Mean ± S.D.	0.93 ± 0.13	0.99 ± 0.06	0.97 ± 0.09	0.067 (kw = 5.416)
	Med (IQR)	1 (0.83–1)	1 (1–1)	1 (1–1)	
Praxis	Mean ± S.D.	0.63 ± 0.2	0.83 ± 0.15	0.84 ± 0.14	0.0001* (kw = 23.253) a, b
	Med (IQR)	0.67 (0.5–0.75)	0.83 (0.75–1)	0.83 (0.75–1)	
Visuomotor Construction	Mean ± S.D.	3.01 ± 1	4.08 ± 0.71	4.18 ± 0.76	0.0001* (kw = 24.743) a, b,c
	Med (IQR)	3.33 (2–3.71)	4.27 (3.5–4.67)	4.42 (3.59–4.83)	
Thinking Operations	Mean ± S.D.	3.13 ± 1.26	4.17 ± 0.78	4.28 ± 0.75	**0.0001** * (kw = 16.533) a, b
	Med (IQR)	3.25 (2–4)	4.25 (3.5–5)	4.5 (3.5–5)	
Memory	Mean ± S.D.	3.08 ± 0.92	3.62 ± 0.59	3.93 ± 0.25	0.0001* (kw = 19.32) b
	Med (IQR)	3 (2.33–4)	4 (3.25–4)	4 (4–4)	
Awareness	Mean ± S.D.	2.33 ± 0.76	3.43 ± 0.68	3.9 ± 0.31	0.0001* (kw = 50.49) a, b,c
	Med (IQR)	2 (2–3)	4 (3–4)	4 (4–4)	

a: Significant difference between cognitive impairment group and Parkinson control group; b: Significant difference between cognitive impairment group and Healthy control group; c: Significant difference between Parkinson control group and Healthy control group

*S.D* Standard Deviation, *Med(IQR)* Median (25th − 75th percentiles), *kw* Kruskal-Wallis Variance Analysis, *z* Mann-Whitney U test

*p < 0.05 statistically significant

The total SMMSE score showed statistically significant moderate positive correlations with orientation, praxis, visuomotor construction, and thinking operations scores across all three groups (respectively; PD-CI group (*r* = 0.651), PD-NCI group (*r* = 0.440), and healthy control group (*r* = 0.640)) (Table 3).

**Table 3:** The relationship between SMMSE total score and DLOTCA-G sub-scale scores of the groups

SMMSE Total Score		PD-CI	PD-NCI	Healthy control group
Orientation	r	**0.651** ^*^	**0.440** ^*^	**0.640** ^*^
	p	0.0001	0.015	0.0001
Visual perception	r	0.287	0.160	0.287
	p	0.125	0.398	0.124
Spatial Perception	r	0.335	-0.358*	0.388^*^
	p	0.071	0.05	0.034
Praxis	r	**0.401** ^*^	**0.417** ^*^	**0.534** ^*^
	p	0.034	0.022	0.002
Visuomotor Construction	r	**0.606** ^*^	**0.494** ^*^	**0.612** ^*^
	p	0.0001	0.005	0.0001
Thinking Operations	r	**0.588** ^*^	**0.425** ^*^	**0.531** ^*^
	p	0.001	0.019	0.003
Memory	r	0.287	0.308	0.380^*^
	p	0.124	0.098	0.039
Awareness	r	0.376^*^	0.239	0.374^*^
	p	0.041	0.204	0.010

*p < 0.05 statistically significant correlation; r: Spearman correlation coefficient

Bold values indicate correlation coefficients of at least moderate strength (*r* ≥ 0.40)

Domain-level analyses in the Parkinson’s disease groups (*n* = 60) showed significant correlations between several SMMSE subdomains and DLOTCA-G subscales. SMMSE Orientation was strongly associated with DLOTCA-G Orientation, Visuomotor Construction, and Awareness (all *p* < 0.001). SMMSE Attention/Calculation and Language showed moderate correlations with executive-related domains, particularly Praxis and Visuomotor Construction (all *p* ≤ 0.001) (Table 4).

**Table 4:** Correlation between SMMSE subscales and DLOTCA-G subscales in Parkinson’s disease groups

DLOTCA-G Subscales		Orientation	Visual Perception	Spatial Perception	Praxis	Visuomotor Construction	Thinking Operations	Memory	Awareness
SMMSE Orientation	r	0.814*	0.340*	0.414*	0.466*	0.661*	0.553*	0.397*	0.665*
	p	< 0.001	0.008	< 0.001	< 0.001	< 0.001	< 0.001	0.002	< 0.001
SMMSE Registration	r	0.113	0.181	-0.058	-0.080	0.132	0.107	0.199	-0.012
	p	0.388	0.166	0.660	0.545	0.315	0.418	0.128	0.928
SMMSE Attention/Calculation	r	0.499*	0.104	0.196	0.471*	0.408*	0.366*	0.381*	0.541*
	p	< 0.001	0.428	0.134	< 0.001	0.001	0.004	0.003	< 0.001
SMMSE Recall	r	0.383*	0.244	0.102	0.305*	0.390*	0.225	0.087	0.240
	p	0.003	0.060	0.439	0.018	0.002	0.084	0.509	0.065
SMMSE Language	r	0.516*	0.408*	-0.020	0.492*	0.550*	0.424*	0.109	0.471*
	p	< 0.001	0.001	0.879	< 0.001	< 0.001	< 0.001	0.407	< 0.001

*p < 0.05 statistically significant correlation; r: Spearman correlation coefficient

Bold values indicate correlation coefficients of at least moderate strength (*r* ≥ 0.40)

## Discussion

This study appears to be the first to evaluate the DLOTCA-G in individuals with Parkinson’s disease in relation to cognitive impairment. Mild cognitive impairment and Parkinson’s disease dementia are the most common nonmotor symptoms of Parkinson’s disease. Mild cognitive impairment associated with Parkinson’s disease has an indistinct onset and progresses gradually. Identifying cognitive deficits at an early stage in Parkinson’s disease plays a vital role in tracking disease progression and planning effective rehabilitation interventions [20].

The results indicate that all subscales of DLOTCA-G, except spatial perception, were negatively affected in individuals with Parkinson’s disease with cognitive impairment. Awareness and visuomotor construction were significantly impaired in patients without cognitive impairment compared to the healthy control group. These findings suggest that DLOTCA-G may be effective in reflecting cognitive involvement in early-stage Parkinson’s Disease, it should be noted that since our study is a cross-sectional one and more research is needed for collecting longitudinal data. Also, a clear relationship was observed between the total SMMSE score and the subscales of orientation, praxis, visuomotor construction, and thinking operations in patients with Parkinson’s disease and healthy elderly individuals. The SMMSE is commonly used in routine practice as a simple, time-efficient, and easy-to-administer cognitive screening test [21]. It primarily assesses orientation, memory, and language skills, and lacks distinct subscales. Visuospatial ability is evaluated in only one subscale, and the test does not cover visuomotor construction or thinking operations processes differentiating. Results can be affected by an individual’s education level, which may lead to false-positive or false-negative results. Early-stage cognitive impairment in patients might go undetected using the SMMSE [22]. Additionally, it should be noted that the SMMSE has limited sensitivity to executive dysfunction, which is a core cognitive domain affected in Parkinson’s disease.

Impairment of visual and spatial functions in Parkinson’s Disease patients can negatively affect their activities of daily living [23]. Studies have reported that visual and spatial function impairments in people with Parkinson’s disease can occur in 20–30% of cases, not only in late-stage patients but also in early-stage and even newly diagnosed patients [24, 25]. Our study demonstrated that several SMMSE subscales particularly Orientation, Visuomotor Construction, and Awareness were significantly correlated with executive-related DLOTCA-G subscales in Parkinson’s disease groups. SMMSE Attention/Calculation and Language showed moderate associations with praxis and visuomotor subscales. These findings show that the SMMSE does capture certain aspects of executive and visuomotor functioning. However, the pattern of these correlations may suggest that DLOTCA-G could offer a more differentiated characterization across multiple cognitive domains, although direct comparisons of discriminative validity remain necessary to substantiate this interpretation. It should be acknowledged that these domain-level analyses are based on correlational associations rather than direct comparisons of discriminative ability between SMMSE and DLOTCA-G subscales. Therefore, the relative sensitivity of DLOTCA-G compared to SMMSE should be interpreted with caution, and future studies employing discriminative analyses are warranted to formally establish this comparison.

Results revealed that healthy older individuals showed impairments in tasks such as copying geometric shapes, sustaining the integrity of objects, and ordering them according to spatial coordinates, even when given unlimited time. Therefore, it has been emphasized that spatial perception may also be adversely affected in healthy older individuals [26]. The lack of significant differences between patients with cognitive impairment, those without cognitive impairment, and the healthy geriatric control group in our study may be due to spatial perception being affected across all three groups but since this was out of scope to our study; without solid evidence and data we must underline that this is a hypothetical conclusion and more research is needed on this conclusion. Regardless of the cause this subscale seems to be less sensitive to early cognitive changes of Parkinson’s disease. It is essential to establish cut-off values for the DLOTCA-G in healthy older individuals to provide guidance for future studies.

Visuomotor construction assesses a patient’s ability to convert perceptual input into a motor response within a spatial context. Motor impairments in Parkinson’s disease can significantly affect neuropsychological test scores, especially those related to visuomotor construction [27]. However, the homogeneous distribution of Hoehn and Yahr staging in our study allowed for an objective evaluation of visuomotor construction scores in relation to motor symptoms. In our study, disease severity was controlled by including only patients in Hoehn and Yahr stages 1–2.5, corresponding to mild-to-moderate Parkinson’s disease. These stages are characterized by preserved functional independence and absence of severe motor disability. This methodological approach reduced the potential confounding effect of advanced motor impairment on task performance. Although bradykinesia and tremor are inherent features of Parkinson’s disease, restricting the sample to these stages lead to a relatively homogeneous motor severity profile. Therefore, the observed differences in visuomotor construction and praxis are more likely to reflect disease-related cognitive and visuospatial dysfunction rather than being solely attributable to motor impairment.

Research has shown that visuomotor control issues, along with disorders of awareness, are early indicators of cognitive impairment in people with Parkinson’s disease who do not have dementia [5]. Research also shows that cognitive impairments in patients with mild cognitive impairment are not primarily related to memory but are more pronounced in areas such as awareness, language, and executive functions [28]. Postural instability is one of the first symptoms in Parkinson’s disease [29]. Visual-motor stimuli are known to be the most preferred means for maintaining balance in patients with Parkinson’s disease [30]. We must underline the fact that these parameters were not tested in our study but they can be impaired even in patients without cognitive impairment [31]. Consequently, hypothetically postural instability may affect the visuomotor construction in people with Parkinson’s disease without cognitive impairment. Numerous studies have reported that awareness and visuomotor construction are significantly more affected in patients with cognitive impairment [32, 33].

Executive functions comprise a heterogeneous group of cognitive abilities, including anticipation, planning, initiation, and monitoring of goal-directed behaviors. Impairment in executive functions leads to significant problems in the daily activities of patients with Parkinson’s disease and may be one of the earliest signs of cognitive impairment [34]. Our study found a significant correlation between total SMMSE scores and several executive function subscales of the DLOTCA-G, which backs the argument that we can effectively evaluate complex and formal executive functions. Some SMMSE subscales showed moderate correlations with executive-related DLOTCA-G subscales, indicating partial overlap between the two instruments. However, because DLOTCA-G uses performance-based and task-oriented tasks, it provides a more detailed and subscale-specific assessment of executive and visuomotor functions. These outcomes suggest that DLOTCA-G offers a broader, clinically meaningful evaluation framework beyond the detection of impairments missed by the SMMSE. Inclusion of DLOTCA-G, which assesses functional and dynamic cognitive domains not captured by SMMSE, strengthens the clinical and ecological relevance of our findings. It is important to note that DLOTCA-G is a multidimensional cognitive battery, not a dedicated executive function test. The executive-related interpretations in this study are based on subscales that partially involve executive processes, such as Thinking Operations, Visuomotor Construction, Praxis, and Awareness, and should not be considered pure executive measures. Executive functions are responsible for regulating the ability to think and act, as well as higher-level cognitive abilities such as abstract reasoning. These abilities can also be affected early in the natural ageing process [35].

Additionally, disorders related to the extrapyramidal system may be observed in normal ageing [36]. The moderate correlation between total SMMSE and similar subscales of the DLOTCA-G in the healthy control group may hypothetically be attributed to declines in high-level cognitive abilities and to potential negative effects on the extrapyramidal system associated with normal ageing, but without solid evidence to back this indirect conclusion this hypothesis needs support and may be a starting point for future research. Multidomain instruments have been developed to aid the detection of cognitive impairment in PD, including tools that integrate cognitive and social functioning domains, PD-specific multidomain rating scales, and measures that evaluate the functional impact of cognitive decline. Future developments in multidomain assessment and early detection of functional decline will be important for advancing early identification strategies [37–39].

Lastly it should be noted that although DLOTCA-G shows potential in assessing Parkinson’s Disease patients for cognitive impairment and shortens the administration time compared to comprehensive neuropsychological batteries; its feasibility in Parkinson clinics with high patient flow considering it requires approximately 50 min to administer, remains a valid question, assessment tools requiring relatively less time like Montreal Cognitive Assessment tool may be a practical alternative for daily clinical use but we believe the outcomes of a DLOTCA-G tests give us more data to modify the rehabilitation plan and to focus to the areas in need of intervention. Also, DLOTCA-G has another advantage as a clinical assessment tools; since it comes with a software that calculates the score automatically, it eliminates largely the human error factor that can be seen in high patient flow clinics while calculating scores. Assessment tools like DLOTCA-G needs scientific visibility to be optimized and researched, thus we think our research contribute to the awareness on that we need more research on this area. Particularly a head-to-head comparison of DLOTCA-G and MoCA for assessing cognitive impairment in Parkinson’s Disease would be a rational step to take for future researchers.

This study has certain limitations. Firstly, participants were grouped only by total SMMSE scores. This approach may lead to methodological circularity and fail to properly reflect PD-MCI, as the SMMSE has limited sensitivity to executive function deficits. In addition, the SMMSE is not highly specific for detecting mild dementia. The absence of a comprehensive neuropsychological gold standard for group classification is a primary limitation. To address this issue in future studies, alternative cognitive screening tools, such as the MoCA or the MDS-UPDRS cognitive subscale, could be used, as they are sensitive to subtle cognitive changes in Parkinson’s disease and can be administered independently of group assignment [40, 41]. Additionally, although ANCOVA was used to control for age and education at the group level, correlation analyses between SMMSE and DLOTCA-G subscales were not adjusted for these covariates via partial correlation methods. Given that both age and educational level may influence cognitive performance, future studies should employ partial correlation approaches to provide more precise estimates of the domain-specific relationships between these instruments. Furthermore, although Hoehn and Yahr staging provided a general index of motor disease severity, detailed motor symptom quantification using the MDS-UPDRS motor subscale was not incorporated into the present analyses. Subtle motor symptoms such as bradykinesia and tremor, even within mild-to-moderate H&Y stages, may have partially influenced performance on motor-dependent DLOTCA-G subscales (particularly Visuomotor Construction and Praxis). Future studies integrating MDS-UPDRS motor scores are recommended to more precisely disentangle motor and cognitive contributions to task performance.

## Conclusion

In conclusion, our findings suggest that DLOTCA-G subscales related to executive processes may be affected in individuals with Parkinson’s disease even in the absence of SMMSE-defined cognitive impairment. While these cross-sectional observations point to the potential value of DLOTCA-G for providing a differentiated cognitive profile, the psychometric properties of DLOTCA-G in Parkinson’s disease—including its diagnostic accuracy, discriminative validity, and comparative sensitivity against MoCA and comprehensive neuropsychological batteries—require formal evaluation in future studies using longitudinal designs and gold-standard reference measures. 

## Data Availability

The datasets used and/or analyzed during the current study are available from the corresponding author on reasonable request.

## Abbreviations

DLOTCA-G: Dynamic Lowenstein Occupational Therapy Cognitive Assessment-Geriatric Version
LOTCA-G: Loewenstein Occupational Therapy Cognitive Assessment-Geriatric
MoCA: Montreal Cognitive Assessment
SMMSE: Standardized Mini-Mental State Examination
MMSE: Mini-Mental State Examination
PD: Parkinson's Disease
PD-CI: Parkinson’s Disease With Cognitive Impairment
PD-NCI: Parkinson's Disease Without Cognitive Impairment

## References

[1] von Campenhausen S, Bornschein B, Wick R, Bötzel K, Sampaio C, Poewe W, et al. Prevalence and incidence of Parkinson’s disease in Europe. Eur Neuropsychopharmacol. 2005;15(4):473–90. 10.1016/j.euroneuro.2005.04.007 15963700

[2] Orgeta V, McDonald KR, Poliakoff E, Hindle JV, Clare L, Leroi I. Cognitive training interventions for dementia and mild cognitive impairment in Parkinson’s disease. Cochrane Database Syst Rev. 2020;2(2):CD011961. 10.1002/14651858.CD011961.pub2 PMC7043362 32101639

[3] Caviness JN, Driver-Dunckley E, Connor DJ, Sabbagh MN, Hentz JG, Noble B, et al. Defining mild cognitive impairment in Parkinson’s disease. Mov Disord. 2007;22(9):1272–7. 10.1002/mds.21453 17415797

[4] Lee HM, Koh SB. Many faces of Parkinson’s disease: non-motor symptoms of Parkinson’s disease. J Mov Disord. 2015;8(2):92–7. 10.14802/jmd.15003 PMC4460545 26090081

[5] Aarsland D, Batzu L, Halliday GM, Geurtsen GJ, Ballard C, Ray Chaudhuri K, et al. Parkinson disease-associated cognitive impairment. Nat Rev Dis Primers. 2021;7(1):47. 10.1038/s41572-021-00280-3 34210995

[6] Chen YR, Tan CH, Su HC, Chien CY, Sung PS, Lin TY, et al. Investigating the interaction between neuropsychiatry features and daily activities on social function in patients with Parkinson’s disease with mild cognitive impairment. BJPsych Open. 2022;8(6):e205. 10.1192/bjo.2022.611 PMC9707510 36426564

[7] Demet Y, Erer S, Zarifoglu M, Mustafa B, Karli N, Nevin T. Evaluation the cognitive functions in Parkinson’s disease patients with neuropsychometric tests and to identify its clinical correlates. Nöropsikiyatri Arşivi. 2010;47(1):47-52 .

[8] Litvan I, Goldman JG, Tröster AI, Schmand BA, Weintraub D, Petersen RC, et al. Diagnostic criteria for mild cognitive impairment in Parkinson’s disease: Movement Disorder Society Task Force guidelines. Mov Disord. 2012;27(3):349–56. 10.1002/mds.24893 PMC3641655 22275317

[9] Katz N, Averbuch S, Bar-Haim Erez A. Dynamic Lowenstein Occupational Therapy Cognitive Assessment–Geriatric Version (DLOTCA–G): assessing change in cognitive performance. Am J Occup Therapy. 2012;66(3):311–9. 10.5014/ajot.2012.002485 22549596

[10] Katz N, Bar-Haim Erez A, Livni L, Averbuch S. Dynamic lowenstein occupational therapy cognitive assessment: evaluation of potential to change in cognitive performance. Am J Occup Therapy. 2012;66(2):207–14. 10.5014/ajot.2012.002469 22394530

[11] Kim J, Shim J-K, Song JW, Kim E-k, Kwak YL. Postoperative cognitive dysfunction and the change of regional cerebral oxygen saturation in elderly patients undergoing spinal surgery. Anesth Analgesia. 2016;123(2):436–44. 10.1213/ANE.0000000000001352 27285000

[12] Ertan T, Eker E, Şar V. Geriatrik depresyon ölçeğinin Türk yaşlı nüfusunda geçerlilik ve güvenilirliği. Nöropsikiyatri arşivi. 1997;34(2):62–71.

[13] Folstein MF, Folstein SE, McHugh PR. Mini-mental state: a practical method for grading the cognitive state of patients for the clinician. J Psychiatr Res. 1975;12(3):189–98. 10.1016/0022-3956(75)90026-6 1202204

[14] Molloy DW, Alemayehu E, Roberts R. Reliability of a standardized mini-mental state examination compared with the traditional mini-mental state examination. Am J Psychiatry. 1991;148(1):102–5. 10.1176/ajp.148.1.102 1984692

[15] Güngen C, Ertan T, Eker E, Yaşar R, Engin F. [Reliability and validity of the standardized mini mental state examination in the diagnosis of mild dementia in Turkish population]. Turk Psikiyatri Derg. 2002;13(4):273–81. 12794644

[16] Crum RM, Anthony JC, Bassett SS, Folstein MF. Population-based norms for the mini-mental state examination by age and educational level. JAMA. 1993;269(18):2386–91. 8479064

[17] Keskinoglu P, Ucku R, Yener G, Yaka E, Kurt P, Tunca Z. Reliability and validity of revised Turkish version of mini mental state examination (rMMSE-T) in community-dwelling educated and uneducated elderly. Int J Geriatr Psychiatry. 2009;24(11):1242–50. 10.1002/gps.2252 19337986

[18] Goldman JG, Holden S, Ouyang B, Bernard B, Goetz CG, Stebbins GT. Diagnosing PD-MCI by MDS Task Force criteria: how many and which neuropsychological tests? Mov Disord. 2015;30(3):402–6. 10.1002/mds.26084 PMC4357536 25449653

[19] Schober P, Boer C, Schwarte LA. Correlation coefficients: appropriate use and interpretation. Anesth Analgesia. 2018;126(5):1763–8. 10.1213/ANE.0000000000002864 29481436

[20] Tan W, Xie F, Zhou J, Pan Z, Liao M, Zhuang L. Efficacy and safety of acupuncture therapy for neuropsychiatric symptoms among patients with Parkinson’s disease: A systematic review and meta-analysis. Clin Rehabil. 2024;38(8):1044–62. 10.1177/02692155241258278 PMC11348633 38840478

[21] Razani J, Wong JT, Dafaeeboini N, Edwards-Lee T, Po L, Alessi n. Predicting everyday functional abilities of dementia patients with the mini-mental state examination. J Geriatr Psychiatr Neurol. 2009;22(1):62–70. 10.1177/0891988708328217 PMC2679691 19196632

[22] Kim HM, Nazor C, Zabetian CP, Quinn JF, Chung KA, Hiller AL, et al. Prediction of cognitive progression in Parkinson’s disease using three cognitive screening measures. Clin Parkinsonism Relat Disorders. 2019;1:91–7. 10.1016/j.prdoa.2019.08.006 PMC7197868 32368733

[23] Williams-Gray CH, Evans JR, Goris A, Foltynie T, Ban M, Robbins TW, et al. The distinct cognitive syndromes of Parkinson’s disease: 5 year follow-up of the CamPaIGN cohort. Brain. 2009;132(11):2958–69. 10.1093/brain/awp245 19812213

[24] Monastero R, Cicero CE, Baschi R, Davì M, Luca A, Restivo V, et al. Mild cognitive impairment in Parkinson’s disease: the Parkinson’s disease cognitive study (PACOS). J Neurol. 2018;265(5):1050–8. 10.1007/s00415-018-8800-4 29478221

[25] Miyasaki JM, Shannon K, Voon V, Ravina B, Kleiner-Fisman G, Anderson K, et al. Practice parameter: evaluation and treatment of depression, psychosis, and dementia in Parkinson disease (an evidence-based review): [RETIRED]. Neurology. 2006;66(7):996–1002. 10.1212/01.wnl.0000215428.46057.3d 16606910

[26] Harada CN, Natelson Love MC, Triebel KL. Normal Cogn Aging Clin Geriatric Med. 2013;29(4):737–52. 10.1016/j.cger.2013.07.002 PMC4015335 24094294

[27] Nieto-Escamez F, Obrero-Gaitán E, Cortés-Pérez I. Visual dysfunction in Parkinson’s disease. Brain Sci. 2023;13(8):1173. 10.3390/brainsci13081173 PMC10452537 37626529

[28] Petersen RC, Smith GE, Waring SC, Ivnik RJ, Tangalos EG, Kokmen E. Mild cognitive impairment: clinical characterization and outcome. Arch Neurol. 1999;56(3):303–8. 10.1001/archneur.56.3.303 10190820

[29] Palakurthi B, Burugupally SP. Postural instability in Parkinson’s disease: a review. Brain Sci. 2019;9(9):239. 10.3390/brainsci9090239 PMC6770017 31540441

[30] Azulay JP, Mesure S, Amblard B, Pouget J. Increased visual dependence in Parkinson’s disease. Percept Mot Skills. 2002;95(3suppl):1106–14. 10.2466/pms.2002.95.3f.1106 12578250

[31] Vaugoyeau M, Azulay J-P. Role of sensory information in the control of postural orientation in Parkinson’s disease. J Neurol Sci. 2010;289(1):66–8. 10.1016/j.jns.2009.08.019 19748102

[32] Burn DJ, Rowan EN, Allan LM, Molloy S, O’Brien JT, McKeith IG. Motor subtype and cognitive decline in Parkinson’s disease, Parkinson’s disease with dementia, and dementia with Lewy bodies. J Neurol Neurosurg Psychiatry. 2006;77(5):585–9. 10.1136/jnnp.2005.081711 PMC2117449 16614017

[33] Burn DJ, Rowan EN, Minett T, Sanders J, Myint P, Richardson J, et al. Extrapyramidal features in Parkinson’s disease with and without dementia and dementia with Lewy bodies: a cross-sectional comparative study. Mov Disord. 2003;18(8):884–9. 10.1002/mds.10455 12889077

[34] Levin BE, Llabre MM, Reisman S, Weiner WJ, Sanchez-Ramos J, Singer C, et al. Visuospatial impairment in Parkinson’s disease. Neurology. 1991;41(3):365–9. 10.1212/wnl.41.3.365 2006002

[35] Cohen RA, Marsiske MM, Smith GE. Chapter 10 - Neuropsychology of aging. In: Dekosky ST, Asthana S, editors. Handb Clin Neurol. Elsevier; 2019;167:149–80. 10.1016/B978-0-12-804766-8.00010-8 31753131

[36] Odenheimer G, Funkenstein HH, Beckett L, Chown M, Pilgrim D, Evans D, et al. Comparison of neurologic changes in ‘Successfully Aging’ persons vs the total aging population. Arch Neurol. 1994;51(6):573–80. 10.1001/archneur.1994.00540180051013 8198468

[37] Kulisevsky J, Fernández de Bobadilla R, Pagonabarraga J, Martínez-Horta S, Campolongo A, García-Sánchez C, et al. Measuring functional impact of cognitive impairment: validation of the Parkinson’s disease cognitive functional rating scale. Parkinsonism Relat Disord. 2013;19(9):812–7. 10.1016/j.parkreldis.2013.05.007 23773412

[38] Yu YW, Tan CH, Su HC, Chien CY, Sung PS, Lin TY, et al. A new instrument combines cognitive and social functioning items for detecting mild cognitive impairment and dementia in Parkinson’s disease. Front Aging Neurosci. 2022;14:913958. 10.3389/fnagi.2022.913958 PMC9243636 35783135

[39] Serrano-Dueñas M, Serrano M, Villena D, Granda D. Validation of the Parkinson’s disease-cognitive rating scale applying the movement disorder society task force criteria for dementia associated with Parkinson’s disease. Mov Disord Clin Pract. 2017;4(1):51–7. 10.1002/mdc3.12338 PMC6174395 30363353

[40] Nasreddine ZS, Phillips NA, Bédirian V, Charbonneau S, Whitehead V, Collin I, et al. The montreal cognitive assessment, MoCA: a brief screening tool for mild cognitive impairment. J Am Geriatr Soc. 2005;53(4):695–9. 10.1111/j.1532-5415.2005.53221.x 15817019

[41] Goetz CG, Tilley BC, Shaftman SR, Stebbins GT, Fahn S, Martinez-Martin P, et al. Movement disorder society‐sponsored revision of the Unified Parkinson’s Disease Rating Scale (MDS‐UPDRS): scale presentation and clinimetric testing results. Mov disorders: official J Mov Disorder Soc. 2008;23(15):2129–70. 10.1002/mds.22340 19025984

