# Correction: the role of dynamic Lowenstein occupational therapy and cognitive assessment scale for the elderly with Parkinson’s disease

**DOI:** 10.1186/s12877-026-08589-y

**Published:** 2026-11-06

**Authors:** Fatıma Yaman, Fatma Akkoyun Arıkan, Esra Aysu Soylu, Elif Pekşen, Merve Akdeniz Leblebicier, Aysun Özlü, Hasan Hüseyin Gökpınar, Barış Yenen, Vural Kavuncu

**Affiliations:** 1 Department of Physical Medicine and Rehabilitation, Kütahya Health Sciences University, Kütahya, Türkiye; 2 Department of Neurology, Kütahya Health Sciences University, Kütahya, Türkiye

Correction: BMC Geriatr 26, 1130 (2026)

https://doi.org/10.1186/s12877-026-07844-6

Following publication of the original article [1], the authors requested to correct the following backmatter sections.

1) Funding section: Replace “None to declare.” with “This work has been supported by Kutahya Health Sciences University Scientific Research Projects Coordination Unit under grant number TSA-2022-93.”

2) Acknowledgements: Replace the sentence “The authors thank the Scientific Research Department of Kütahya Health Sciences University for providing support throughout the study period (Project ID: TSA-2021-24).” with “The authors thank the Scientific Research Projects Coordination Unit of Kütahya Health Sciences University for providing support throughout the study period (Project ID: TSA-2022-93).”

3) Ethics approval and consent to participate: Delete the final sentence “The DLOTCA-G evaluation battery was provided as part of a scientific research project affiliated with the Kütahya Health Sciences University Scientific Research Department (Project Code: TSA-2021-24).”

The original article [1] has been updated.
